# Effects of Video-Based Visual Training on Decision-Making and Reactive Agility in Adolescent Football Players

**DOI:** 10.3390/sports4010001

**Published:** 2015-12-31

**Authors:** Alfred Nimmerichter, Nikolaus J. R. Weber, Klaus Wirth, Andreas Haller

**Affiliations:** Sport and Exercise Sciences, University of Applied Sciences Wiener Neustadt, Johannes Gutenberg Str. 3, Wiener Neustadt A-2700, Austria; nikolaus.weber@wvnet.at (N.J.R.W.); klaus.wirth@fhwn.ac.at (K.W.); andreas.haller@fhwn.ac.at (A.H.)

**Keywords:** visual training, video-based, decision-making, reactive agility, anticipation, one-on-one, transfer effect, football

## Abstract

This study investigated the trainability of decision-making and reactive agility via video-based visual training in young athletes. Thirty-four members of a national football academy (age: 14.4 ± 0.1 years) were randomly assigned to a training (VIS; *n =* 18) or a control group (CON; *n =* 16). In addition to the football training, the VIS completed a video-based visual training twice a week over a period of six weeks during the competition phase. Using the temporal occlusion technique, the players were instructed to react on one-on-one situations shown in 40 videos. The number of successful decisions and the response time were measured with a video-based test. In addition, the reactive-agility sprint test was used. VIS significantly improved the number of successful decisions (22.2 ± 3.6 s *vs.* 29.8 ± 4.5 s; *p* < 0.001), response time (0.41 ± 0.10 s *vs.* 0.31 ± 0.10 s; *p =* 0.006) and reactive agility (2.22 ± 0.33 s *vs.* 1.94 ± 0.11 s; *p =* 0.001) pre- *vs.* post-training. No significant differences were found for CON. The results have shown that video-based visual training improves the time to make decisions as well as reactive agility sprint-time, accompanied by an increase in successful decisions. It remains to be shown whether or not such training can improve simulated or actual game performance.

## 1. Introduction

Perceptual-cognitive skills such as anticipation and decision-making are crucial performance determinants in team sports such as football, where fast and accurate decisions are required in a complex and rapidly changing environment [1]. Players must pick up information from the ball, teammates and opponents, and the decision-making process occurs under pressure with opponents trying to restrict “time” and “space” available [2]. To initiate quick actions, athletes must focus their attention and visual perception on the most relevant information sources or key events [3]. This is particularly important considering that athletes are constantly confronted with an abundance of environmental and contextual information in every situation.

A number of studies investigated perceptual abilities of high performance athletes and it was shown that there are fundamental perceptual and cognitive differences between experts and novices [4,5,6]. For instance, better tactical predictions [7], higher anticipation precision [8], and improved situational adaption of eye movement patterns [9] among the experts have been reported. Casanova *et al.* [10] identified four perceptual-cognitive abilities to illustrate the perceptive superiority of experts compared to novices. These are the ability of players to quickly deduce game-relevant key characteristics (advanced cue utilization), knowledge of situations specific to the sport (situational probabilities), recognition of movement patterns (pattern recognition) and visual search strategy. These qualities are also associated with “game intelligence” [11]. Perceptual-cognitive skills have been described as “executive functions”, regulating thought and action, and have been suggested to predict the success of ball sport players [12]. Sheppard *et al.* [13] emphasized the importance of perceptive performance for agility, which is a decisive skill in football. In addition, skills such as anticipation, decision-making, reactivity and creativity are considered key elements of reactive agility [11,14].

Eye-tracking studies have been shown that experts possessed a superior perceptual system when compared to non-experts. For instance, analysis of visual search behavior found that experts had more visual fixations of a shorter duration, recognized game scenes quicker, localized relevant objects more precisely and anticipated actions of their opponents earlier [5,15]. The results of the visual movement tracking show that the complexity of situations in game sports requires a deliberate focus of visual attention just on game-relevant information.

Numerous studies addressed the training modalities of perceptual-cognitive skills. Research into video training over the past two decades focused on perceptual-cognitive skills in team sports [16,17,18,19,20] as well as in individual sports [4,8,21]. Sport-specific, video-based perception training has been confirmed to improve perceptual and cognitive skills (for an overview, see [22,23]). The goal of this form of visual perception training is to accelerate the process of performance development, bringing the perceptual abilities of a novice up to expert level as quick as possible [24]. Video-based studies frequently use the temporal occlusion technique [3,8,25,26,27] where sport-specific video sequences are interrupted at a certain time, leaving subjects to anticipate the actual outcome of the scene. It has been shown that the perspective of the videos shown can affect the learning rate and, therefore, such videos are typically presented from the “first-person” perspective (*i.e.*, perspective of the player) [28].

Although video-based training has been shown to improve perceptual skills, studies on the transferability to sport-specific performance are scarce. Therefore, the aims of this study were to investigate the effects of video-based visual training on decision-making and the transfer to a reactive-agility sprint test in youth football players. It was hypothesized that video-based visual training will improve decision-making and reactive agility sprint-time.

## 2. Method

### 2.1. Participants

A total number of 36 male, trained participants from a national football academy were recruited to participate in the study, which was conducted during the competition phase. Two participants from the control group withdrew and thus 34 players completed the study. The players had a training history of 3–5 years and trained 7–10 h·week^−1^. The performance level can be considered high, as the players competed in the highest Austrian youth league and some of them were part of the junior national team. The study was conducted in accordance with the ethical principles of the Declaration of Helsinki and approved by the institutional review board. The participants and their parents were briefed as to the risks of the study and provided written informed consent to participate.

### 2.2. Study Design

Participants were matched in accordance with their age category (*i.e.*, U15 and U16) and assigned to either the training group (VIS; *n =* 18; mean ± SD age: 14.4 ± 0.4 years; stature: 171.2 ± 7.2 cm; body mass: 61.8 ± 8.3 kg) or the control group (CON; *n =* 16; mean ± SD age: 14.4 ± 0.5 years; stature: 169.5 ± 7.8 cm; body mass: 59.7 ± 8.1 kg). In addition to their regular football training, the VIS group completed a six-week intervention program consisting of video-based visual training performed twice a week. The visual training consists of a series of 5–8 s one-on-one video sequences and requires the players to react in response to the video scene (described below). Before and after the training intervention participants were asked to complete a video-based test to assess anticipation and decision-making. In addition, reactive agility was measured using the sprint test described by Sheppard *et al.* [13]. Participants completed two familiarization days before the study, including all training and testing procedures.

### 2.3. Construction of Training Videos

Prior to commencement, 12 amateur football players were asked to record video sequences of one-on-one situations with a helmet camera (GoPro HD Hero 2; GoPro Inc., San Mateo, CA, USA) from the first-person perspective of the forward. The videos were recorded at a resolution of 1280 × 960 pixels at 48 frames per second and a viewing angle of 170 degrees. The horizontal and vertical image area enables the capturing of both the defending player and the ball.

The attacking behavior of the defenders was pre-arranged and consisted of the four most common defending tackles. These consisted of block tackling, sliding tackling and both front- and back-foot-side tackling, and were chosen on the basis of a video analysis of various national and international football matches.

Before each recording, the defenders were instructed in which way to tackle the forward and to execute the movement in a realistic, dynamic posture to mimic a real one-on-one situation as accurately as possible. Furthermore, defenders were asked to run toward the forward, match his speed and finally attack him. It was also permitted to use feints, but eventually the pre-arranged tackle must be executed.

From a total number of 800 recorded videos, 456 videos were pre-selected and categorized into one of the four defending tackles. Each of the four categories therefore contained 114 videos and the duration of each video was about 5–8 s. To improve the quality of the recordings, video sequences were edited and blurred recordings were stabilized. From every category 20 videos were excluded to be used for the pre- and post-test and were not included in the training intervention. The remaining video sequences were used for the training group during video-based visual training.

### 2.4. Training Intervention

Over a period of six weeks, two video-based training sessions per week were completed immediately before regular football training between 17 and 19 h. Each training consisted of 32 randomly presented video sequences (eight per defending category) and lasted ~6 min. The videos were individually presented in quiet rooms adjacent to the changing rooms on four laptops (Lenovo ThinkPad T420s) using a commercially available video player (VLC Media Player).

The videos were prepared using the temporal occlusion technique. This time-sensitive sealing technique stops the video at fixed times, in such a way that only certain elements can be perceived, but not the whole movement. The attention should be drawn toward the most informative stimulus, in order to identify the actions of the opponent as soon as possible and optimize their own reaction time.

Using this technique, a 2 s pause was added at the initiation of the defense. During this brief interruption, the participants were asked to mark either “left” or “right” on a prepared form, indicating the direction they believed the forward could successfully pass the defender. After the pause, the video resumed and subjects were made aware of the outcome of the one-on-one situation, thus receiving feedback on their own response. If no answer was marked during the standstill, the answer was considered false.

### 2.5. Computer Test

The video-based test consisted of 40 videos (10 per defending category), which were shown at the same time of day and place as the training videos and lasted ~12 min. As described above, these videos were exclusively used for the video-based test and not during the training intervention. The videos were prepared with a specialized video player (Utilius^®^ fairplay 5, CCC Software GmbH, Leipzig, German) to include a time marker at the moment the defender initiated his defensive tackle. This software contains a stopwatch function with an accuracy of 0.01 s. Additionally, the software enabled smooth playback of HD video recordings and could pause precisely at the push of a button.

In contrast to the training videos, test videos were not paused, but subjects stopped the video sequences by pushing a button, along with a stopwatch running in the background. Immediately after stopping the video, subjects had to shout out “left” or “right”, again indicating the direction they believed the forward could successfully pass the defender. The direction as well as the time the video was stopped was noted in a prepared computer test report by the supervisor. The number of successful decisions (*i.e.*, direction) and the temporal delay between the time marker and the moment the video was stopped were used to assess the effects of the video-based training on decision-making and anticipation.

### 2.6. Reactive-Agility Sprint Test

**Figure 1:**
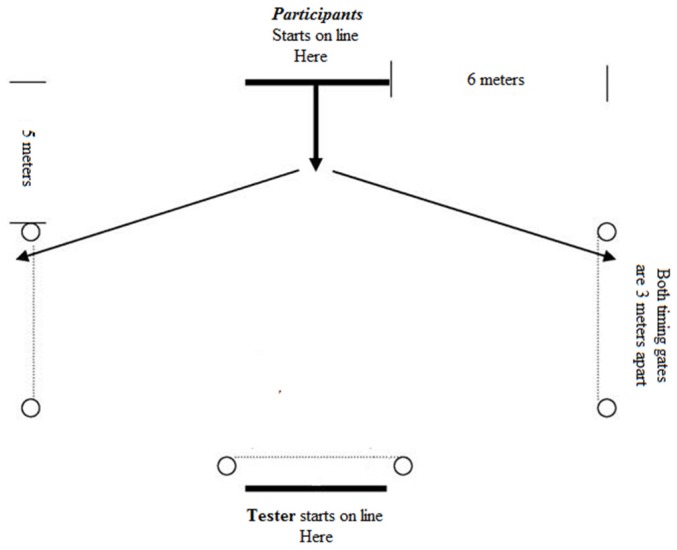
Schematic description of the reactive-agility sprint test [13].

To assess agility in response to a stimulus, the reactive-agility sprint test was used, as it has been shown to provide reliable and valid results. The test-retest intraclass correlation coefficient (ICC) was 0.878 and the technical error of measurement 0.005 s [13]. In the present study the test was conducted on a football field on artificial turf at sea level, at a temperature of 17° and at a wind speed <2 m∙s^−1^ using timing gates (Brower TC Timing Systems; Draper, UT, USA) (Figure 1).

After a standardized warm-up of 15 min, subjects started on the mark opposite to the supervisor and were asked to react as quickly as possible to a tackling stimulus induced by the supervisor by sprinting either to the left or right. The timing was started by the supervisor and stopped by the player by sprinting through the left or right timing gate. Each participant completed six sprints and the average of the sprint times was used to assess reactive agility.

### 2.7. Statistical Analyses

Statistical analyses were performed with the software SPSS Statistics 21 (IBM Corporation; Armonk, NY, USA). Descriptive data are reported as mean ± standard deviation (SD). After the assumption of normality was verified using the Shapiro-Wilk test, a two-factorial repeated measure analysis of variance with group as the between (VIS *vs.* CON) and time (pre *vs.* post) as the within factor was used to assess the effect of the intervention on response time, response accuracy and sprint time. Significant main effects were followed up with Bonferroni *post-hoc* tests for pairwise comparisons. The level of significance was set at *p* < 0.05.

## 3. Results

### 3.1. Computer Test

**Figure 2:**
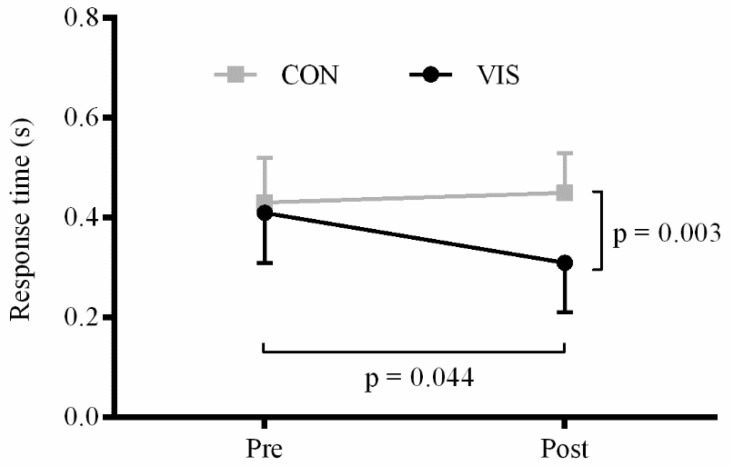
Response time (s) obtained during the computer test. *P*-values for the main effects of time and group are shown. Interactions and *post-hoc* results are presented in the text.

*Response time:* There were significant main effects of time (F_1,32_ = 4.4; *p =* 0.044) and group (F_1,32_ = 10.3; *p =* 0.003). In addition, a significant time × group interaction was observed (F_1,32_ = 9.8; *p =* 0.004). The training group significantly improved response time from 0.41 ± 0.10 to 0.31 ± 0.10 s (*p =* 0.006) whereas no significant difference was observed for the control group (0.43 ± 0.09 *vs.* 0.45 ± 0.08 s; *p =* 0.297) (Figure 2).

**Figure 3:**
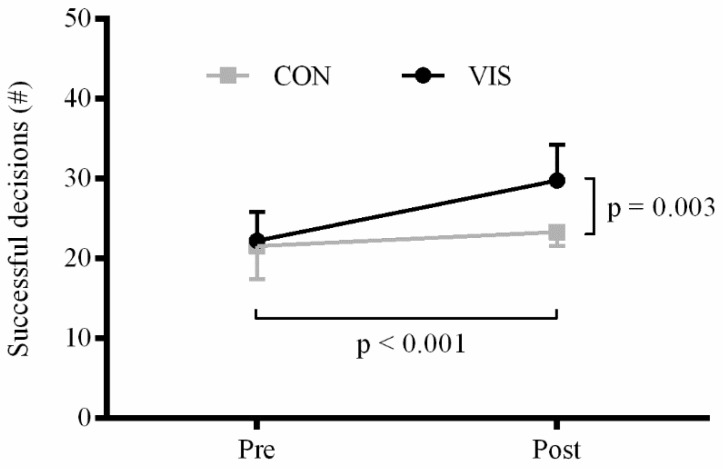
Response accuracy obtained during the computer test. *P*-values for the main effects of time and group are shown. Interactions and *post-hoc* results are presented in the text.

*Response accuracy:* There were significant main effects of time (F_1,32_ = 32.9; *p* < 0.001) and group (F_1,32_ = 10.0; *p =* 0.003). In addition, a significant time × group interaction was observed (F_1,32_ = 12.8; *p =* 0.001). The training group significantly improved the number of successful decisions from 22.2 ± 3.6 to 29.8 ± 4.5 (*p* < 0.001) whereas no significant difference was observed for the control group (21.6 ± 4.2 *vs.* 23.3 ± 3.8; *p =* 0.125) (Figure 3).

### 3.2. Reactive-Agility Sprint Test

**Figure 4:**
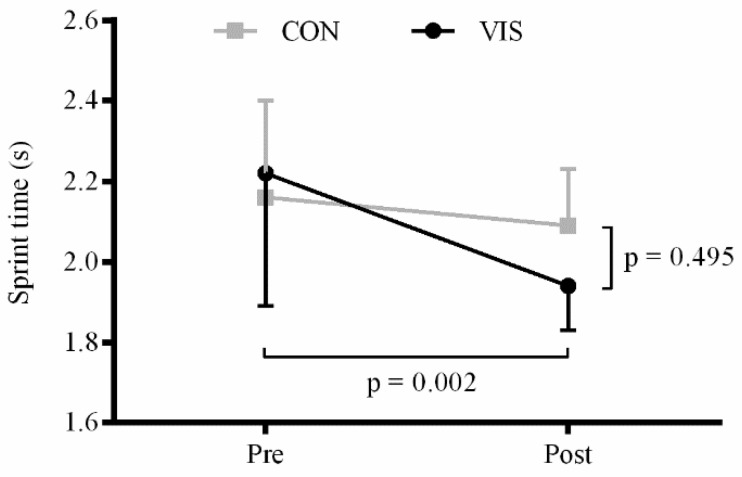
Sprint time (s) obtained during the reactive-agility sprint test. *P*-values for the main effects of time and group are shown. Interactions and *post-hoc* results are presented in the text.

*Sprint time:* There was a significant main effect of time (F_1,32_ = 12.1; *p =* 0.002) but not of group (F_1,32_ = 0.5; *p =* 0.495). A significant time × group interaction was observed (F_1,32_ = 4.4; *p =* 0.043). The training group significantly improved the sprint time from 2.22 ± 0.33 to 1.94 ± 0.11 s (*p =* 0.001) whereas no significant difference was observed for the control group (2.16 ± 0.24 *vs.* 2.09 ± 0.14 s; *p =* 0.363) (Figure 4).

## 4. Discussion

The aim of this study was to examine the effects of video-based visual training on decision-making and reactive agility in adolescent football players. The main findings were that the visual training group significantly improved response time and response accuracy during the computer test, as well as sprint time obtained from the reactive-agility sprint test. These results suggest that video-based visual training is an appropriate stimulus to train the perception-action coupling in young trained football players.

A number of studies reported differences in perceptual and cognitive skills between experts and novices [4,5,6] and there is little doubt that experts extract and use the information available (*i.e.*, opponents movement pattern) faster than less-experienced athletes. There is also some evidence of improvements in response time and response accuracy after video-based perceptual training [22,23]. However, there is limited evidence on the transferability into sport performance [25].

The participants in the present study were recruited from a national football academy, had a training history of 3–5 years and underwent the same training during the six-week intervention. The visual training group significantly improved the response time during the computer test by 24% (0.10 s), whereas no significant change ( −5% or 0.02 s) was observed in the control group. This result is a 31% difference between the training and the control group after the intervention. Considering the importance of fast decision-making in team sports such as football, this improvement could be a decisive advantage. In addition to fast decision-making, football also requires accurate decisions. In the present study, faster response times did not have negative effects on response accuracy. The visual training group significantly improved the number of successful decisions by 34% (7.56). A small but non-significant change of 8% (1.75) was also found in the control group, resulting in a 28% difference between the training and the control group after the intervention. It could be argued that in a two-choice prediction task (*i.e.*, “left” or “right”), any changes might be the result of guessing. A success rate of approximately 50% (20 successful decisions in the present study) could be expected if a participant is only guessing. However, all but one (–1) participant of the visual training group improved response accuracy. Twelve subjects increased the correct answers by more than five and seven subjects by more than 10 correct answers. In contrast, 10 subjects of the control group improved response accuracy, only two by more than five correct answers. We are therefore confident that our findings are, at least in part, the result of the video-based training.

In addition to the computer-based test, the present study also employed a reactive-agility sprint test to assess the transfer to a field-based performance measure. In accordance with the computer-based test, the visual training group was able to significantly improve sprint time by 13% (0.28 s), while an insignificant change of 3% (0.07 s) was found in the control group. Our results support previous studies in field hockey [29] and rugby [16], showing a transfer from video-based training to field performance. The field test utilized in this study used a semi-specific trigger signal (*i.e.*, tackling stimulus) to induce a more realistic, game-like situation compared with reactive sprint tests that utilize sounds or lights as trigger signals. Given the importance of perception for reactive-agility in football [13], the improvement in reactive-agility sprint time suggests, although does not prove, that video-based training can positively affect game performance. However, reactive-agility is only one of many fitness components in football, thus indicating the complex nature of competitive performance [30]. It should be noted that the participants in our study continued with regular football training during the six-week intervention. However, as the players were from the same national academy and underwent the same training regime, we do not assume a specific effect of athletic training on sprint performance in the visual training group. In addition, the randomization of the groups would minimize such an effect.

In contrast to other studies [17,20,28], where more complex game situations were used to evaluate decision-making, the present study used one-on-one situations recorded from a first-person perspective. The videos were recorded with a helmet camera worn by the forward and therefore represent a dynamic “self-perception” of the playing action. Self-perception has been described as key to mimic the playing action as realistically as possible and to obtain valid and transferable performance effects [28,31]. In accordance with the present study, Hagemann *et al.* [17] found significant improvements in perception-action coupling in three *versus* two game situations.

The training intervention in the present study was conducted over a six-week period, commenced twice a week, and each training session last approximately six minutes. In comparison, previous studies on video-based training used training durations between 10 min and two hours, over time periods up to six weeks [7,16]. It remains to be shown whether or not an increase in training duration and/or number of training sessions could further improve perceptual skills or enhance the transfer into field performance. For example, Williams and Grant [7] reported more pronounced performance improvements in the initial phase compared to the later phase of a video-training intervention. In addition, the longitudinal effects after training cessation need to be further elucidated. Currently, only a few studies reported retained effects in decision-making after two weeks [32] and five months [33], while others did not find such effects after a 32-day retention period [25]. As mentioned above, the participants in our study continued with regular football training during the intervention and it is currently unclear whether or not a video-based visual training alone (e.g., off-season or injury) would improve perceptual-cognitive skills. The efficacy of such training alone without imposing physical strain would further strengthen its practical application.

The study is not without limitations. Firstly, although two familiarization days before the study were conducted to reduce learning effects, we cannot totally exclude such effects associated with the tests. Secondly, this was the first study employing the video-based computer test and, therefore, the test-retest reliability is currently unknown. Thirdly, to increase the ecological validity of the findings, further studies are required to investigate the effects of video-based visual training with the occurrence of fatigue during simulated or actual game situations.

## 5. Conclusions

In conclusion, the present study showed that video-based visual training results in faster and more accurate decisions during a computer-based test and improves sprint times obtained from a reactive-agility sprint test. It has been shown that youth football players can improve perceptual-cognitive skills with a 6 min video-based training performed twice a week. Therefore, video-based visual training is considered an effective supplement to regular football training. However, further studies are required to investigate the effects in elite-level players, the long-term effects and the efficacy of such training during periods where no specific football training is scheduled.
